# New synthesis and biodistribution of the D-amino acid oxidase-magnetic nanoparticle system

**DOI:** 10.4155/fso.15.67

**Published:** 2015-09-11

**Authors:** Francesca Cappellini, Camilla Recordati, Marcella De Maglie, Loredano Pollegioni, Federica Rossi, Marco Daturi, Rosalba Gornati, Giovanni Bernardini

**Affiliations:** 1 Dipartimento di Biotecnologie e Scienze della Vita, Università degli Studi dell'Insubria, Via Dunant 3, Varese, Italy; 2 Mouse & Animal Pathology Laboratory, Fondazione Filarete, Viale Ortles, Milano, Italy; 3 Dipartimento di Scienze Veterinarie e Sanità Pubblica, Università degli Studi di Milano, Via Celoria, Milano, Italy; 4 The Protein Factory, Politecnico di Milano and Università degli Studi dell'Insubria, Via Mancinelli 7, Milano, Italy; 5 Laboratoire Catalyse et Spectrochimie, ENSICAEN, Universit' de Caen, CNRS, 6 Bd Maréchal Juin, F-14050 Caen, France

**Keywords:** anticancer system, bionanoparticles, *in vivo* analysis, iron, IR spectroscopy, nanoenzyme, nanoparticles

## Abstract

**Background:**

Application of nanoenzymes, based on D-amino acid oxidase (DAAO) conjugated to magnetic nanoparticles (NPs), as anticancer system requires improvement of the synthesis protocol and *in vivo* distribution evaluation.

**Results:**

A new and more efficient synthesis via EDC-NHS produced an Fe_3_O_4_NP-APTES-DAAO system with a specific activity of 7 U/mg NPs. IR spectroscopy showed that all Fe_3_O_4_ NP sites are saturated with APTES and all available NH_2_ sites with DAAO. The acute cytotoxicity of the new system does not differ from that of the previous one. *In vivo* experiments showed that the system did not cause adverse effects, cross the brain–blood barrier and accumulate in the heart.

**Conclusions:**

Our results support the possibility to use enzymes conjugated to magnetic NPs for cancer treatment. Besides, we think that enzymes and other biological molecules efficiently conjugated to magnetic NPs might constitute a category of ‘bionanoparticles’ to be exploited, not only in medical, but also in industrial biotechnology.

## Abstract (lay abstract)

**Lay abstract:** We have linked magnetic nanoparticles to D-amino acid oxidase, an enzyme capable of producing, in the presence of its substrate, reactive oxygen species. The scope is to use the magnetic properties of the enzyme-nanoparticle system to direct it to a desired area where its cytotoxicity can be controlled by the addition of exogenous substrate. Besides the possible applications in cancer therapy, we think that enzymes and other biological molecules linked to magnetic nanoparticles might also be exploited in industrial biotechnology.

**Figure 1.**
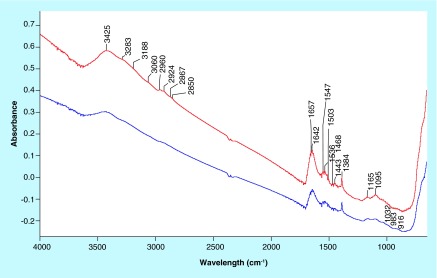
IR spectra of Fe_3_O_4_-APTES-DAAO prepared via glutaraldehyde (red) and via EDC/NHS (blue). Samples were dispersed in KBr (2% w/w).

**Figure 2.**
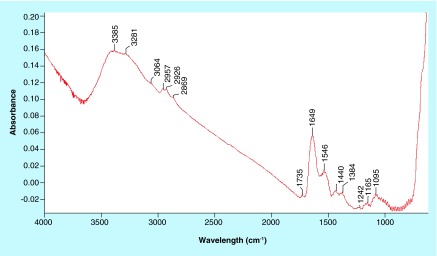
IR spectral subtraction. Spectrum of Fe_3_O_4_-APTES-DAAO prepared with the first method minus those prepared with the second method.

**Figure 3.**
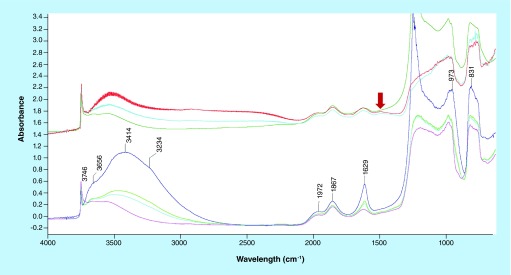
IR spectra of samples dispersed in silica. Bottom: in blue, subtraction of glutaraldehyde and EDC/NHS samples, in cyan, magenta and green spectra of glutaraldehyde samples after NO and CO adsorption. Top: EDC/NHS samples spectra after CO adsorption. Arrow indicates peak at 1508 cm^-1^.

**Figure 4.**
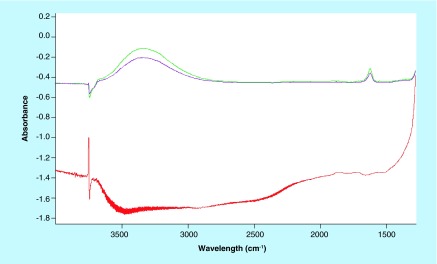
Spectra in the top refer to NO adsorption (10 and 18 torr at the equibrium) on the glutaraldehyde sample. Spectrum in the bottom is the difference before and after the adsorption of CO on the EDC/NHS sample.

**Figure 5.**
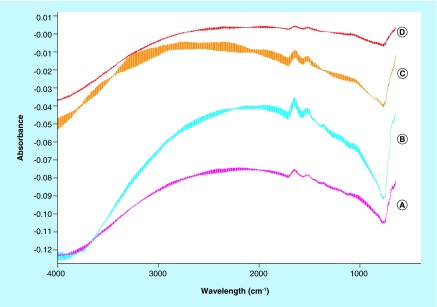
Spectra of samples collected on a silicon wafer. A and B refer to glutaraldehyde samples while C and D refers to EDC/NHS samples.

**Figure 6.**
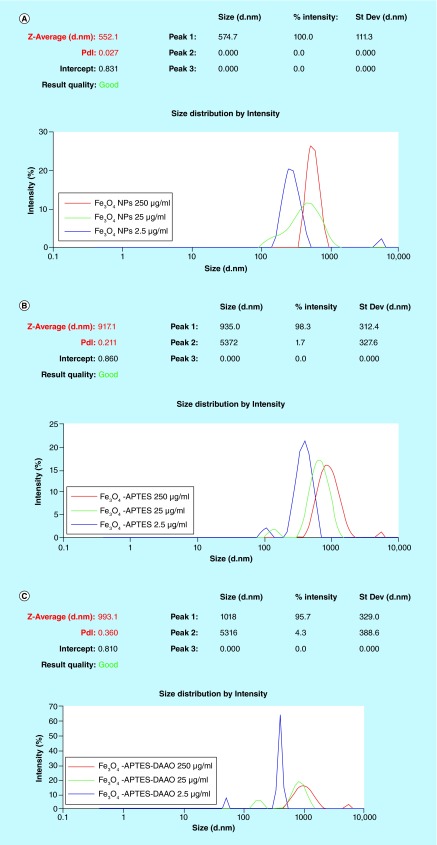
DLS analysis on Fe_3_O_4_ NPs **(A)**, Fe_3_O_4_-APTES **(B)** and Fe_3_O_4_-APTES-DAAO **(C)** at different concentrations.

**Figure 7.**
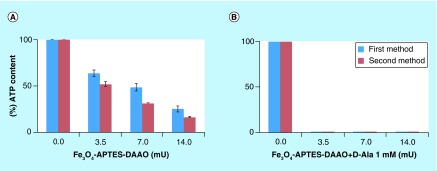
Comparison of cytotoxicity, on SKOV-3, of Fe3O4-APTES-DAAO prepared with the two methods, without and with 1 mM D-Ala.

**Figure 8.**
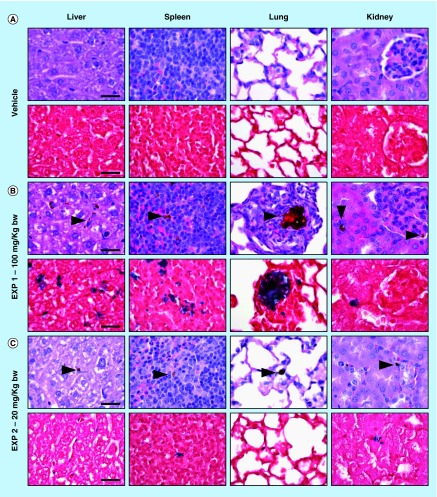
Histology of liver, spleen, lung, and kidney of mice treated with vehicle **(A)**, 100 mg/kg (**[B]**; EXP 1), and 20 mg/kg (**[C]**; EXP 2) of NPs (upper panel: HE; lower panel: Perls stain; scale bar = 25 µm). Brown granular material is evident in HE stained sections (arrowheads) of NP treated mice, consistent with iron aggregates, as confirmed by Perls stain (blue).

**Figure 9.**
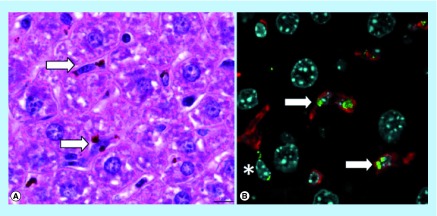
Histological examination of mouse liver. (A) Brown aggregates of iron-containing NPs within the cytoplasm of Kupffer cells lining hepatic sinusoids (HE staining). (B) Iron-containing NPs (green) are mainly found in the cytoplasm of Iba1+ Kupffer cells (red, arrows) and occasionally in spindled cells (*). Nuclei stained with DAPI (cyan).

**Figure 10.**
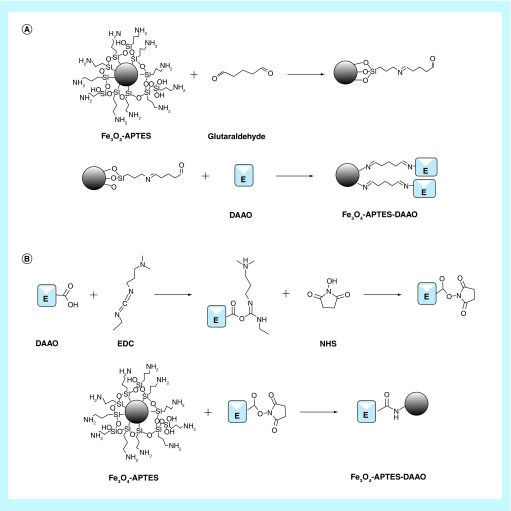
Schematic representation of the two DAAO conjugation methods on Fe_3_O_4_-APTES: **(A)** Conjugation via glutaraldehyde; **(B)** Conjugation via EDC and NHS.

Nanoparticles (NPs) and other nanomaterials permeate several areas of our everyday life. In industrial applications, they have become indispensable components of catalysts [1], sensors [2] and photovoltaic devices [3]. Unfortunately, the same peculiar properties that make nanomaterials so attractive may create potentially new and largely undefined risks for human health. NPs, in effect, are capable to cross biological barriers [4–6] and are readily taken up by cells [7] where they can exert their toxicity. However, NPs have also found widespread use in the biomedical field, as nanovaccines, nanodrugs and diagnostic imaging tools [8]. The increasing number of nanotechnology patents related to healthcare reflects the rapid expansion of this pioneering industry [9].

The impact of nanobiotechnology on oncology is huge and includes fields ranging from discovery of tumor biomarkers to development of devices for cancer surgery [10,11]. In particular, drug-delivery, the most explored field, aims to design carriers that deliver drugs more precisely to tumor cells and maintain them at therapeutic concentrations over a long period [12]. Besides considerable progress in cancer therapy and the many anticancer drugs available, the treatment of cancer has still a lot of side effects [13] and NPs could be an efficient alternative to conventional therapies, serving as carrier systems capable of enhancing efficacy, while simultaneously reducing side effects. Magnetic NPs can be functionalized with anticancer drugs and guided along an externally placed magnet into the tumor [14]. Moreover, magnetic NPs can produce heat through various energy losses under an external alternating magnetic field, causing cancer destruction by hyperthermia [15–20].

In a previous work [21], we set up the synthesis of an NP-enzyme system for cancer therapy via glutaraldehyde. The system (Fe_3_O_4_-APTES-DAAO) was based on iron oxide magnetic NPs conjugated to the ROS-producing enzyme D-amino acid oxidase from *Rhodotorula gracilis* (RgDAAO, EC 1.4.3.3). Fe_3_O_4_-APTES-DAAO aims to combine the advantages of magnetic NPs (low acute toxicity, specific target by a magnetic field, ability to cross biological barriers) with those of RgDAAO (no acute toxicity, easy modulation of the activity, hence of ROS generation). The system can be intravenously injected and addressed by an external magnetic field in the tumor area, where D-amino acids (naturally present or externally injected) act as substrate for the enzyme, causing H_2_O_2_ production and tumor cell death by apoptosis.

This work is on continuing the research of using magnetic NPs conjugated to DAAO for the potential treatment of cancer, a step forward in the possible realization of this clinical technique. To this aim, we present a new and more efficient synthesis of this system via EDC-NHS and the characterization by infra red (IR) spectroscopy of the two systems resulting from the old and the new methods of synthesis. Moreover, we have analyzed the *in vivo* distribution as well as the acute toxicity of the new system after single intravenous administration in mice. We have also realized that enzymes conjugated to magnetic NPs could be of interest also for industrial purposes [22].

## Materials & methods

### Coating of magnetic NPs with APTES

Fe_3_O_4_ NPs were functionalized according to the protocol of Bava *et al.* [21]. Briefly, 150 mg of Fe_3_O_4_ NPs (Sigma, cat. number 637,106) were ultrasonicated in 10 ml of H_2_O MilliQ for 15 min. A solution of 5 ml APTES (2% v/v, Sigma cat. number A3648) in H_2_O MilliQ was then added and the reaction was maintained under mechanical stirring for 5 h at 50°C. Fe_3_O_4_-APTES were then separated from unbound APTES by a commercial parallelepiped neodymium magnet (Webcraft GmbH, Uster, Switzerland; Ni-Cu-Ni plated; magnetization: N45; size: 30 × 30 × 15 mm), washed several times with water and anhydrificated with ethanol overnight. Fe_3_O_4_-APTES were suspended again in water, ultrasonicated for 30 min and left at room temperature for 1 h, isolated and dried at 50°C overnight.

### Synthesis of Fe_3_O_4_NP-APTES-DAAO

Four milligrams of Fe_3_O_4_NP-APTES were suspended by sonication for 15 min with 1-ethyl-3-(3-dimethylaminopropyl)carbodiimide (EDC, Sigma cat. number 03450) and N-hydroxysuccinimide (NHS, Sigma, cat. number 130,672) in 3:2 (w/w) ratio in 5 mM sodium pyrophosphate (NaPPi) buffer, pH 8.5. Two hundred and fifty micrograms of recombinant RgDAAO [19] were added and the reaction was carried out for 4 h at 4°C using a rotating plate tube stirrer. Subsequently, Fe_3_O_4_-APTES-DAAO were collected by a magnet and washed twice with 1 ml of 5 mM NaPPi pH 8.5. The supernatant was stored for further analysis.

### DAAO activity assay

The activity of Fe_3_O_4_-APTES–DAAO was determined by measuring the absorbance increase accompanying the H_2_O_2_-induced oxidation of *o*-dianisidine [20]. One DAAO unit corresponds to the amount of enzyme that converts 1 μmol of substrate per min at 25°C and at 0.253 mM oxygen concentration [23,24]. The standard assay mixture contained 890 μl of 100 mM D-alanine in 100 mM NaPPi buffer, pH 8.5 buffer, 100 μl of 3.2 mg/ml *o*-dianisidine in water, 10 μl of 0.4 mg/ml horseradish peroxidase in 100 mM NaPPi buffer, pH 8.5 buffer and 10 μl of 0.4 mg/ml Fe_3_O_4_NP-APTES-DAAO in the same buffer. The reaction was initiated by the addition of the enzyme and the absorbance increase was monitored at 440 nm for 1 min using an UV-Vis Jasco V-560 spectrophotometer.

### IR analysis

Fe_3_O_4_NP-APTES-DAAO were characterized by IR analysis in different conditions. Samples (disks of 2 cm^2^ area) were placed in a quartz cell equipped with KBr windows. A movable quartz sample holder allows to adjust the pellet in the infrared beam for spectra recording and to displace it into a furnace at the top of the cell for thermal treatment. The cell was connected to a vacuum line for evacuation (P_residual_ = ˜10^–6^ torr) and for the introduction of gases into the infrared cell. Spectra were recorded at room temperature. The addition of well-known doses of gas in the cell was possible via a pressure gauge for the control of the gas pressure. A Nicolet Nexus spectrometer equipped with a Mercury Cadmium Telluride (MCT) cryodetector and an extended KBr beam splitter was used for the acquisition of spectra in the 600–5500 cm^-1^ range. IR spectra are absorption spectra and the notation used is a.u. for absorption units. The resolution of the spectra was 4 cm^-1^, and 256 scans were accumulated for each spectrum.

Three kinds of experiments have been performed: first the samples have been dispersed in the KBr, then on the surface of a silica pellet; finally on a silicon disk. In details: a wafer containing 98 mg of KBr has been pressed at 4 tons/cm^2^, together with ˜2 mg of the targeted sample; a wafer of 20 mg silica has been casted mixing in its center ˜6–7 mg of sample, then pressed to 2 tons/cm^2^ and ˜2 mg of powder have been dispersed on the surface of a 2 cm^2^ silicon disk by the help of a spot of ethanol.

### Dynamic light scattering (DLS) analysis

Fe_3_O_4_, Fe_3_O_4_-APTES and Fe_3_O_4_-APTES-DAAO NPs were analyzed at concentrations of 250, 25 and 2.5 μg/ml in water. Sample dilutions were done in order to analyze the effect of concentration on NP aggregation. Each measurement was preceded by an equilibration time of 90 s. Analyzes were performed with Zetasizer Nano ZS90 (Malvern, UK) instrument. Measures were reported as scattering intensity in function to diameter.

### Cell viability

SKOV-3 cell lines were maintained as adherent cells in RPMI1640 medium (supplemented with 10% fetal bovine serum, 1% L-glutamine and 1% penicillin/streptomycin solution) at 37°C in a humidified 5% CO_2_ atmosphere. Cells were passaged as needed using 0.25% trypsin–EDTA.

Cell viability was determined as ATP content by using the CellTiter-Glo Luminescent Cell Viability Assay according to the manufacturer's instruction. In details, 1 × 10^4^ cells were seeded into 96-well assay plates and cultivated for 24 h at 37°C in 5% CO_2_ to equilibrate and become attached prior to the treatment. Cells were then exposed to increasing amounts of Fe_3_O_4_-APTES-DAAO, in presence or not of its substrate, for 24 h in a final volume of 100 µl. Following the treatment, plates were equilibrated for 30 min at room temperature and 100 µl of CellTiter-Glo Reagent was then added to each well. Plates were shaken for 2 min and left at room temperature for 10 min prior to the recording of luminescent signals using the Infinite F200 plate reader (Tecan Group, Männedorf, Switzerland). The experiments were performed in triplicate. Cell viability, expressed as ATP content, was normalized against control values.

### *In vivo* studies

To evaluate the biodistribution and acute toxicity of NPs, two *in vivo* studies were performed. In both experiments, male CD-1(ICR) mice of 4–5 weeks were purchased from Charles River (Calco, Italy). They were acclimated to the environment for a week prior to the initiation of the study, with free access to water and a standard pellet diet *ad libitum*. The environmental conditions were set at a temperature of 22 ± 2°C, relative humidity of 55 ± 10% and a 12 h light:dark cycle. The mice (n = 3) were intravenously injected with a single dose of Fe_3_O_4_-APTES-DAAO (in the first experiment 100 mg/kg bodyweight (bw) of NPs in NaCl 0.9%, and in the second experiment 20 mg/kg bw of NPs in NaPPi buffer at pH 7.4) and sacrificed 24 h after the treatment. The control groups were treated with the corresponding vehicle (NaCl 0.9% or NaPPi buffer). Immediately after the treatment and the following hours, the general health and behavior of mice were monitored. The bodyweight of each mouse was measured before treatment and at sacrifice. The experiment was performed in accordance with the Italian Laws (D.L. 116/92 and following additions), which enforce EU 86/609 Directive (Council Directive 86/609/EEC of 24 November 1986, on the approximation of laws, regulations and administrative provisions of the member states regarding the protection of animals used for experimental and other scientific purposes).

### Sacrifice, sampling & pathological examination procedures

Mice were euthanized at 24-h post-treatment according to standard procedures and in compliance with local regulations, and underwent complete necropsy. Liver, spleen and kidney were weighed and the relative organ weight (wet organ/total bodyweight) was calculated.

Liver, kidney, spleen, lung, heart, testis and brain were fixed in 10% neutral-buffered formalin, and routinely processed for paraffin embedding. Four micrometer sections from each tissue were stained with hematoxylin and eosin (HE) and Perls iron stain [25], and evaluated under a light microscope. Quantitative evaluation of iron deposits in Perls iron stained sections was performed by digital image analysis: area of Perls staining was measured in three 200× microscopic fields using the ImageJ analysis program [26], and the percentage of Perls positive area per field was then calculated.

For immunofluorescence, liver sections were immunostained with rabbit monoclonal anti-Iba1 antibody (Wako Chemicals, VA, USA, cat. number 019–19741), a pan-macrophage marker [27]. Secondary antibody, Alexa Fluor^®^ 555 F(ab')2 Fragment of Goat Anti-Rabbit IgG (H+L; Life Technologies Europe BV, Monza, Italy, cat. number A-21430) was then added. Immunofluorescently labeled sections were acquired with the Leica TCS SP5 confocal microscope (Leica Microsystems GmbH, Wetzlar, Germany). The Alexa555 fluorophore was excited with the 561 nm laser line and the emitted fluorescence (570–700 nm) acquired with a 63×/1.4 oil immersion objective (Leica Microsystems, GmbH). Nuclei were visualized by DAPI staining (405 nm laser line excitation, 415–500 nm acquisition window). Iron aggregates were visualized by reflection of light at 561 nm.

### Statistical analysis

Data were analyzed using GraphPad Prism version 5.0 (GraphPad Software, CA, USA). Statistical analyses of the results were performed using Mann–Whitney U test to compare the experimental conditions with controls. p-values <0.05 were considered statistically significant.

## Results

### Activity analysis

RgDAAO was conjugated to Fe_3_O_4_-APTES by means of EDC and NHS. Under best experimental conditions, the amount of enzyme bound to NPs, determined as the difference between the protein amount added and that recovered in the supernatant, is close to 100%, with an enzymatic activity of approximately 7 U/mg NP.

### IR characterization

The spectra of Fe_3_O_4_-APTES-DAAO prepared with the two methods (the old one *via* glutaraldehyde and the new one presented here), dispersed in the KBr (to identify the structural components), are reported in Figure 1. The two spectra are similar. At the highest wavenumbers, we can observe the stretching of the OH groups of water adsorbed on KBr, likely 3425 cm^-1^. In the 3300–3000 cm^-1^ region, the ν(NH) vibrations are observed; they correspond to the NH_2_ moieties present in the organic ligand. The corresponding bending mode is at 1650–1640 cm^-1^ and 1546 cm^-1^, partially overlapped with the δ(HOH) of water at ˜1630 cm^-1^. Below 800 cm^-1^, only structural (saturated) bands are visible, containing bending, twisting and rocking modes, together with the stretches of M-O bonds. The other bands (in the 1600–1000 cm^-1^ region) are due to C-C and C-O bonds, while those in the 3000–2800 cm^-1^ interval to C-H stretches, difficult to be discriminated at this step. Interestingly, no bands at ˜1745 cm^-1^, typical of the C=O bonds of the aldehydes, can be distinguished. Performing a spectral subtraction (Figure 2) we can better scrutinize the differences in the two compounds: the vibrations associated to the NH_2_ species almost disappear in the compound prepared via EDC/NHS, as indicated by the bands in positive in the difference spectrum (3400–3000 cm^-1^ region, plus 1649 and 1546 cm^-1^ peaks) and by the absence of the corresponding bands in the blue spectrum in Figure 1. A very weak feature appears at 1735 cm^-1^, accounting for aldehyde typical vibration.

When dispersing the samples on silica as a support (Figure 3), the observed bands essentially deal with OH groups on silica (3746 cm^-1^) and adsorbed water (˜3450 and 1630 cm^-1^). The features at 1972 and 1867 cm^-1^ are overtones and combination bands of the silica framework (massif below 1300 cm^-1^) (spectrum blue, Figure 3). The sample has been probed by adsorption of CO and NO, two molecules adapted to reveal (and quantify) the presence of coordinatively unsaturated iron cations [28]. After CO or NO adsorptions, no remarkable differences can be distinguished especially on the glutaraldehyde sample (Figure 3, spectra cyan, magenta and green). In the case of the EDC/NHS sample, a weak component appears at 1508 cm^-1^ (Figure 3, spectrum red, red arrow), which cannot be related directly with the probe molecules. Eventually, it could be ascribed to CO interactions with some accessible ligand in the organic part, inducing a shift in a C–C or C–O vibration. Subtraction spectra (Figure 4) confirm that no effect of probe molecule adsorption can be detected, so suggesting the absence of unsaturated iron moieties.

Spectra of the samples characterized after deposition on a silicon wafer (to discard any potential interaction with a support) are reported in Figure 5. Besides the interference fringes due to the specular surfaces of the Si disks, we can observe only bands due to the organic ligands, already mentioned above. Comparing the spectra during the adsorption of CO as probe molecule and after evacuation (B and A for glutaraldehyde, respectively, and C and D for EDC/NHS), we cannot observe any difference, if we exclude the changes in the baseline, irrelevant for the present study. Even, this latter approach for the sample analysis seems less appropriate, providing spectra of relatively low quality.

### DLS analysis

In all samples, production of NP aggregates of 300 nm or more is evident. The size of the aggregates varies with the dilution: at increased dilution the diameter decreases. Aggregation of metal NPs in aqueous environment has been demonstrated to depend on several factors, concentration included [29]. Moreover, the polydispersity index (pdi) increases from Fe_3_O_4_ NPs to Fe_3_O_4_-APTES-DAAO (Figure 6).

### Cytotoxicity

The cytotoxicity of the two systems was tested on SKOV-3 cells with and without the substrate D-alanine (1 mM). As reported in Figure 7, no significant differences were evident: both systems in the presence of the substrate fully depleted the ATP content at 3.5 mU of DAAO.

### *In vivo* study

#### Mice treated with 100 mg NP/kg

Immediately after the administration of NPs, and during the following hours, all mice appeared healthy and no relevant behavioral alterations were observed. At necropsy, the liver of three out of three treated mice had a diffuse mild black-brown discoloration compared with control mice. No significant differences in bodyweight gain and relative organ weights of liver, spleen and kidney were observed between control and Fe_3_O_4_-APTES-DAAO treated group (Table 1). Histologically, in three out of three treated mice, intracytoplasmic brown granular material (consistent with iron pigment, as confirmed by Perls iron stain) was found in HE stained sections of the liver, spleen and lung, and only occasionally in the kidney (at level of blood vessels) (Figure 8). The results of the quantitative evaluation of the iron deposits detected in Perls iron stained sections of examined organs is reported in Table 2. In the liver the iron pigment was found mainly in the cytoplasm of Kupffer cells (immunostained with Iba1), but occasionally also within spindle-shaped cells, likely consistent with hepatic sinusoidal endothelial cells (Figure 9). In the lung, there were moderate numbers of cells (monocytes/macrophages) with intracytoplasmic iron pigment infiltrating the alveolar septa, throughout the pulmonary parenchyma. Multifocally, intravascular up to 100 µm in diameter iron aggregates (emboli) surrounded by granulocytes, were found in association with interstitial infiltrates of histiocytes expanding the alveolar septa (Figure 8). In the spleen, large numbers of histiocytes with intracytoplasmic iron pigment were found in the red pulp, marginal zone, and PALS/germinal center. The white pulp was affected by mild-to-moderate follicular reactive hyperplasia. No iron deposits were observed in the heart, and brain of treated mice.

#### Mice treated with 20 mg NP/kg

No relevant behavioral alterations and no significant differences of bodyweight gain, and relative organ weights of liver, spleen and kidney were observed between control and Fe_3_O_4_-APTES-DAAO-treated group (Table 1). Grossly, a moderate splenomegaly was observed only in one out of three treated mice. Histologically, in three out of three treated mice, small-to-moderate amount of iron pigment was found in the liver (Kupffer cells), spleen (macrophages/histiocytes in the marginal zone, red pulp and PALS/germinal center) and rarely in the kidney (interstitial capillaries). In one out of three treated mice, only rare and small intracytoplasmic iron aggregates (monocytes/macrophages) were found in the lung alveolar septa (Figure 8). No iron deposits were observed in the heart, testis and brain. Reduced amount of iron-containing deposits were detected in liver and spleen compared with those found in mice treated with 100 mg NP/kg (Table 2).

## Discussion

In our previous work, we designed a protocol for the immobilization of RgDAAO on magnetic iron oxide NPs for antitumor therapy (Fe_3_O_4_-APTES-DAAO) [21]. The conjugation was performed via glutaraldehyde obtaining a system with an activity of 4 U/mg NP. In this work, we improved the protocol for the synthesis of Fe_3_O_4_-APTES-DAAO, enhancing the yield by 40%, reaching 100% of conjugated enzyme in term of activity and quantity. The conjugation of RgDAAO occurs by means of EDC and NHS and the reaction times are reduced by 2 h. The new system has an activity of 7 U/mg NP, 1.5-fold more than the old one (characterized by 4.5 U/mg NP). A schematic representation of the two protocols of conjugation is reported in Figure 10.

The comparison of the two systems by IR analysis demonstrates that all the ligands are functionalized in the EDC-NHS sample, whereas a small portion of them present free coordination ends in the glutaraldehyde compound (Figures 1 & 2). In light of this, we can say that the EDC-NHS sample higher activity in respect with the glutaraldehyde sample is due to a higher efficiency of bound. In fact, apparently all available NH_2_ sites are saturated with DAAO, while glutaraldehyde sample shows some free NH_2_ and aldehyde sites. Moreover all conjugation sites of Fe_3_O_4_ NPs are saturated, signifying that APTES functionalization is absolute.

DLS analysis shows that the population of Fe_3_O_4_-APTES-DAAO is stable around a dimension of 1 μm indicating that the enzyme conjugation process induces aggregation of NPs. However, more information on the hydrodynamic diameter of the iron core, the magnetization capabilities and the iron content would allow to better compare these newly created particles to others.

The cytotoxicity analysis does not highlight any difference between the two systems; indeed, both completely deplete the ATP content at 3.5 mU in the presence of the DAAO substrate D-alanine. Worthy of note is that the second system employs a lower amount of Fe_3_O_4_ NPs, because of the higher enzymatic activity.

In the present paper, we have also evaluated the *in vivo* safety of the Fe_3_O_4_-APTES-DAAO system produced with the EDC-NHS procedure. In the first experiment, after intravenous injection in mice of 100 mg NP/kg diluted in 0.9% NaCl no significant alterations of behavior, bodyweight gain and relative organ weight of liver, spleen and kidney were observed. Histologically, iron deposits were detected in the liver, spleen, lungs and kidneys, while none was found in the heart and brain. Mainly, iron deposits were found in the cytoplasm of monocytes/macrophages/histiocytes, indicating that most of injected particles were removed from blood circulation by phagocytic cells, as previously reported for other nanoparticles [30]. However, presence of intravascular iron deposits associated with interstitial inflammation were found in the lung, indicating that pulmonary embolization occurred after intravenous administration of aggregated NPs, as confirmed by the results of DLS analysis. In the spleen of mice treated with 100 mg/kg, reactive follicular hyperplasia was observed. This finding may be interpreted as an antigenic response, likely directed against the enzymatic portion of the NP system. In general, no overt adverse effects have been observed 24 h after intravenous administration of 100 mg Fe_3_O_4_-APTES-DAAO/kg, however, the presence of pulmonary embolization of iron aggregates prompted us to perform a second experiment using a lower dose (20 mg NP/kg), and a different vehicle (NaPPi buffer instead of 0.9% NaCl) in order to prevent particle aggregation. No clinicopathological adverse effects were observed after intravenous administration of 20 mg NP/kg, and histologically, a dose-dependent decrease of iron deposits was found in the liver and spleen. In addition, absence of detectable iron deposits in the brain and testis (examined only for 20 mg Fe_3_O_4_-DAAO/kg) suggests that our system is not able to cross biological barriers, or, at least, not in the absence of an external magnetic field and under tested conditions.

We also think that biological molecules, such as enzymes, polysaccharides [31,32], antibiotics, antibodies and nucleic acids [33], efficiently conjugated to magnetic NPs might constitute a category of ‘bionanoparticles’ to be exploited in medical as well as industrial biotechnology.

## Conclusion

We have improved the synthesis protocol of Fe_3_O_4_-APTES-DAAO, reaching the highest activity allowed by the system. The acute cytotoxicity of the newly synthesized nanoenzyme does not differ from the previously synthesized one. Moreover, no relevant adverse effects were observed after *in vivo* intravenous administration in mice of a single dose of 20 mg NP/kg bw.

## Future perspective

Future aim will be the investigation of the biodistribution of NPs under application of an external magnetic field. The ability of an external magnetic field to accumulate NPs in a determined area as well as that to make NPs crossing biological barriers will be investigated. The ability of the system to destroy tumors will also be tested *in vivo*.

Moreover, we are convinced that flavoenzymes efficiently conjugated to magnetic NPs could be exploited also in industrial biotechnology [34].

**Table 1.** **Mean bodyweight gain (%) and relative organ weight (%) following intravenous administration of vehicle, 100 and 20 mg/kg bodyweight of nanoparticles in mice.**

**EXP**	**Group**	**Bodyweight gain**	**Liver**	**Spleen**	**Kidney**
1	Vehicle (0.9% NaCl)	-1.6 ± 1.9	6.6 ± 1.2	0.5 ± 0.1	1.9 ± 0.0
	Fe_3_O_4_-DAAO 100 mg/kg	-3.5 ± 2.2	7.8 ± 0.3	0.6 ± 0.1	1.6 ± 0.1
2	Vehicle (NaPPi)	-2.9 ± 0.4	6.7 ± 0.1	0.5 ± 0.1	1.8 ± 0.1
	Fe_3_O_4_-DAAO 20 mg/kg	-3.5 ± 2.8	6.9 ± 0.2	0.5 ± 0.1	1.6 ± 0.2

Results are expressed as means ± SD.

**Table 2.** **Quantitative evaluation of iron deposits detected in Perls iron stained sections.**

**Group**	**Liver**	**Spleen**	**Lung**	**Kidney**	**Brain**	**Heart**
Vehicle	0.02 ± 0.02	0.02 ± 0.00	0.05 ± 0.02	0.04 ± 0.01	0.00 ± 0.00	0.00 ± 0.00
Fe_3_O_4_-DAAO 100 mg/kg	2.98 ± 1.11	1.40 ± 0.55	0.51 ± 0.13	0.09 ± 0.05	0.00 ± 0.00	0.00 ± 0.00
Fe_3_O_4_-DAAO 20 mg/kg	0.23 ± 0.07	0.17 ± 0,06	0.13 ± 0.08	0.04 ± 0.01	0.00 ± 0.00	0.00 ± 0.00

Results are expressed as means ± SD.

**Executive summary**

**Improvement of the synthesis protocol of the system Fe_3_O_4_-APTES-DAAO**

The protocol reached the highest possible efficiency in terms of enzyme immobilization yield and activity and the time of production was reduced by 2 h.

**Characterization of the old & the new system & analysis of in vivo biodistribution**

IR analysis confirmed the absolute saturation of all binding sites. Fe_3_O_4_-APTES-DAAO cannot cross biological barrier without a magnetic field. The intravenous injection of 20 mg Fe_3_O_4_-APTES-DAAO/kg does not cause adverse effects.

**Future perspective**

The capability of the system to cross biological barriers, to be targeted in a specific area and to destroy a tumor will be investigated. Moreover, a possible exploitation of nanoenzymes in industrial biotechnology will be considered.

## References

[1] Astruc D, Astruc D (2008). Transition metal nanoparticles in catalysis: from historical background to the state of the art. *Nanoparticles and Catalysis (Chapter 1)*.

[2] Saha K, Agasti SS, Kim C, Li XN, Rotello VM (2012). Gold nanoparticles in chemical and biological sensing. *Chem. Rev.*.

[3] Stratakis E, Kymakis E (2013). Nanoparticle-based plasmonic organic photovoltaic devices. *Mater. Today*.

[4] Barua S, Mitragotri S (2014). Challenges associated with penetration of nanoparticles across cell and tissue barriers: a review of current status and future prospects. *Nano Today*.

[5] Castellini C, Ruggeri S, Mattioli S (2014). Long-term effects of silver nanoparticles on reproductive activity of rabbit buck. *Syst. Biol. Reprod. Med.*.

[6] Coccini T, Gornati R, Rossi F (2014). Gene expression changes in rat liver and testes after lung instillation of a low dose of silver nanoparticles. *J. Nanomed. Nanotechnol.*.

[7] Papis E, Rossi F, Raspanti M (2009). Engineered cobalt oxide nanoparticles readily enter cells. *Toxicol. Lett.*.

[8] Cattaneo AG, Gornati R, Sabbioni E (2010). Nanotechnology and human health: risks and benefits. *J. Appl. Toxicol.*.

[9] Antunes AMD, Alencar MSD, da Silva CH, Nunes J, Mendes FML (2012). Trends in nanotechnology patents applied to the health sector. *Recent Pat. Nanotechnol.*.

[10] Jain KK (2010). Advances in the field of nanooncology. *BMC Med.*.

[11] Salmasi S, Kalaskar DM, Yoon W-W, Blunn GW, Seifalian AM (2015). Role of nanotopography in the development of tissue engineered 3D organs and tissues using mesenchymal stem cells. *World J. Stem Cells*.

[12] Ediriwickrema A, Saltzman WM (2015). Nanotherapy for cancer: targeting and multifunctionality in the future of cancer therapies. *ACS Biomater. Sci. Eng.*.

[13] Kanwar JR, Sebastian M, Ninan N, Elias E (2013). Nanotechnological based system for cancer. *Nanomedicne and Cancer Therapies (Volume 2)*.

[14] Liberatore M, Barteri M, Megna V (2015). Effect of external magnetic field on IV ^99^mTc-labeled aminosilane-coated iron oxide nanoparticles: demonstration in a rat model: special report. *Clin. Nucl. Med.*.

[15] Lim EK, Jang E, Lee K, Haam S, Huh YM (2013). Delivery of cancer therapeutics using nanotechnology. *Pharmaceutics*.

[16] Gang J, Park SB, Hyung W (2007). Magnetic poly ε- caprolactone nanoparticles containing Fe3O4 and gemcitabine enhance anti-tumor effect in pancreatic cancer xenograft mouse model. *J. Drug Target.*.

[17] Lee JH, Chen KJ, Noh SH (2013). On-demand drug release systems for *in vivo* cancer treatment through self-assembled magnetic nanoparticles. *Angew. Chem. Int. Ed.*.

[18] Cho MH, Lee EJ, Son M (2012). A magnetic switch for the control of cell death signaling in *in vitro* and *in vivo* systems. *Nat. Mat.*.

[19] Thomas CR, Ferris DP, Lee JH (2010). Noninvasive remote-controlled release of drug molecules *in vitro* using magnetic actuation of mechanized nanoparticles. *J. Am. Chem. Soc.*.

[20] Katagiri K, Imai Y, Kounoto K, Kaiden T, Kono K, Aoshima S (2011). Magnetoresponsive on-demand release of hybrid liposomes formed from Fe3O4 nanoparticles and thermosensitive block copolymer. *Small*.

[21] Bava A, Gornati R, Cappellini F, Caldinelli L, Pollegioni L, Bernardini G (2013). D-amino acid oxidase-nanoparticle system: a potential novel approach for cancer enzymatic therapy. *Nanomedicine (London)*.

[22] Netto C, Toma HE, Andrade LH (2013). Superparamagnetic nanoparticles as versatile carriers and supporting materials for enzymes. *J. Mol. Catalysis B Enz.*.

[23] Fantinato S, Pollegioni L, Pilone MS (2001). Engineering, expression and purification of a His-tagged chimeric D-amino acid oxidase from *Rhodotorula gracilis*. *Enzyme Microb. Technol.*.

[24] Harris CM, Pollegioni L, Ghisla S (2001). pH and kinetic isotope effects in d‐amino acid oxidase catalysis. *Eur. J. Biochem.*.

[25] Peophet EB, Prophet EB, Mills B, Arrington JB, Sobin LH (1994). Armed Forces Institute of Pathology Staf. *AFIP Laboratory Methods in Histotechnology*.

[26] ImageJ. http://www.rsb.info.nih.gov/ij/.

[27] Rehg JE, Bush D, Ward JM (2012). The utility of immunohistochemistry for the identification of hematopoietic and lymphoid cells in normal tissues and interpretation of proliferative and inflammatory lesions of mice and rats. *Toxicol. Pathol.*.

[28] Wuttke S, Bazin P, Vimont A (2012). Discovering the active sites for C3 separation in MIL-100(Fe) by using operando IR spectroscopy. *Chem. Eur. J.*.

[29] Keller AA, Wang H, Zhou D (2010). Stability and aggregation of metal oxide nanoparticles in natural aqueous matrices. *Environ. Sci. Technol.*.

[30] Fujihara J, Tongu M, Hashimoto H (2015). Distribution and toxicity evaluation of ZnO dispersion nanoparticles in single intravenously exposed mice. *J. Med. Invest.*.

[31] Bava A, Cappellini F, Pedretti E (2013). Heparin and carboxymethylchitosan metal nanoparticles: an evaluation of their cytotoxicity. *Biomed. Res. Int.*.

[32] Vismara E, Valerio A, Coletti A (2013). Non-covalent synthesis of metal oxide nanoparticle–heparin hybrid systems: a new approach to bioactive nanoparticles. *Int. J. Mol. Sci.*.

[33] Fratila RM, Mitchell SG, del Pino P, Grazu V, de la Fuente JM (2014). Strategies for the biofunctionalization of gold and iron oxide nanoparticles. *Langmuir*.

[34] Pollegioni L, Molla G (2011). New biotech applications from evolved D-amino acid oxidases. *Trends Biotechnol.*.

